# Collaborative Outreach in Ecology Through Social and Natural Science Partnerships in the Classroom

**DOI:** 10.1002/ece3.74197

**Published:** 2026-08-20

**Authors:** Jennifer L. Funk, Carrie A. Furman, Elizabeth M. Becker, Brooke E. Wainwright, Sandra N. Amoh, Sameen Ahmed, Irene A. Darkwaah, Mariana Cubillos, Hannah Jose, Sandra E. Kotoka, Meleanitema Sapoi‐Finau

**Affiliations:** ^1^ Department of Plant Sciences University of California, Davis Davis California USA; ^2^ Emory University Atlanta Georgia USA; ^3^ Department of Biology San Diego State University San Diego California USA

**Keywords:** boundary objects, co‐production, graduate training, qualitative methods, restoration ecology, social‐ecological research

## Abstract

Ecologists increasingly work in multi‐disciplinary projects with communities, land managers, and policy makers to translate ecological knowledge into on‐the‐ground action, yet graduate training rarely provides structured practice in the cross‐disciplinary collaboration that this work demands. We present a case study of a project‐based graduate seminar co‐taught by an ecologist and a social scientist that paired eight social science master's students (Emory University) with two ecology doctoral students (San Diego State University and University of California, Davis) in a semester‐long, largely remote collaboration. Student teams used a research proposal as a boundary object to co‐produce socially‐informed ecological research designs, supported by readings and staged feedback, mid‐semester cross‐team discussion, and final presentations. The collaboration yielded two applied proposals: (1) an assessment framework for an elementary, place‐based science program using pre/post “sticker” surveys, observation, and stakeholder interviews/focus groups; and (2) a mixed‐methods study of how US restoration researchers and practitioners' goals and decision‐making are shifting, including openness to using ecosystem function targets versus historic species composition. Student reflections highlighted several friction points, including translation across disciplinary language, differing methodological instincts and epistemic norms, and virtual coordination constraints, but also reported meaningful gains in mutual respect, clearer communication, and a more realistic understanding of the labor required to collaborate well. We synthesize these lessons into practical recommendations for course design, including early cross‐training, frequent structured touchpoints, iterative co‐generation of questions and methods, and balanced instructor presence.

## Graphical abstract

Ecologists increasingly collaborate with communities, land managers, and policy makers, yet graduate training rarely offers structured practice in the cross‐disciplinary skills this work requires. We present a case study of a project‐based, largely remote graduate seminar co‐taught by an ecologist and a social scientist that paired eight social science master's students with two ecology doctoral students to co‐produce socially informed ecological research proposals using the proposal itself as a boundary object. Student products and reflections reveal both predictable friction points and clear learning gains, which we synthesize into practical recommendations for designing and facilitating interdisciplinary graduate training.

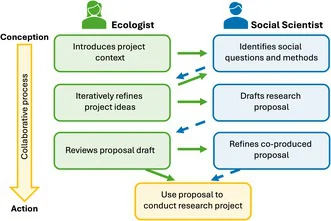

## 1 Introduction

As researchers in an interdisciplinary field, ecologists increasingly work with communities, land managers, and policy makers who rely on them not only to understand ecological processes but also to inform decisions about land use, restoration, climate adaptation, and biodiversity conservation (Bufford et al. 2025; Groffman et al. 2010). These problems are embedded in particular places, histories, and communities, and meaningful progress often depends on the ability of ecologists to work across disciplinary and institutional boundaries while also engaging directly with the people who implement ecological knowledge on the ground (Löfqvist et al. 2022). For example, in many areas of ecology, including invasion ecology, restoration ecology, and conservation biology, a persistent “knowing–doing” gap exists where scientific information accumulates but is inconsistently incorporated into management decisions (Funk et al. 2020; Matzek et al. 2014). In response, researchers in these fields are increasingly working in multi‐disciplinary projects to co‐design studies, test interventions in real‐world settings, and integrate ecological insights into on‐the‐ground action (Gornish et al. 2023; Shackleton et al. 2019).

Social scientists' theories and methods offer powerful tools for addressing implementation and communication challenges. Qualitative approaches such as interviews, focus groups, ethnography, and participant observation, along with mixed‐methods survey and participatory techniques, can illuminate how people experience environmental change, how institutions shape management decisions, and what motivates or constrains the uptake of ecological recommendations (e.g., Barak et al. 2022; Funk et al. 2014; Furman et al. 2018; Rowe 2010; Stern 2018). Nonetheless, ecological research still favors quantitative, biophysical approaches, while qualitative methods remain misunderstood, underutilized, and sparsely cited (Anderson et al. 2021). Expanding opportunities for ecologists to learn, practice, and appreciate the complexity of social science methods is essential for extending ecological knowledge and improving policy, management, and conservation outcomes (Bennett et al. 2017). However, introducing these approaches to early‐career ecologists remains uncommon, as explicit training in interdisciplinary collaboration and social science methods is still limited in many graduate pathways (Kelly et al. 2019; Record et al. 2016).

In this paper, we present a case study of one such opportunity: a project‐based graduate seminar that brought together students from the natural and social sciences to learn how to design collaborative, community‐engaged ecological research. The seminar was grounded in the idea that if ecological research aims to generate socially relevant outcomes, social scientists and community‐engaged approaches should be integrated early, during problem framing and study design, rather than added only after key research decisions have already been made. Co‐taught by an ecologist and a social scientist, the course introduced students to qualitative and mixed‐methods approaches, guided them in translating ecological research questions for broader audiences, and supported the development of community‐oriented research proposals. Rather than reporting on the implementation of those projects, we focus on the seminar as a model for graduate training in interdisciplinary research design and proposal development. Specifically, we describe the course structure, the collaborative process, the products students developed, and lessons learned for institutions seeking to prepare social and natural scientists to work together in service of more effective ecological practice. A companion paper will provide a more detailed account of student reflections on interdisciplinary collaboration, including their expectations, challenges, frustrations, and strategies for working across disciplinary boundaries (Furman et al., unpublished data).

## 2 Curriculum Design and Aims

Our goal in developing this class‐based project was to give students a realistic experience of collaborating with colleagues outside their home discipline, including noticing how needs and expectations can differ and how much institutional context or background knowledge often has to be shared to work effectively. Through that collaboration, students were challenged to learn the methods, language, and working processes characteristic of other fields, including the practical work of translating terms and concepts across disciplinary boundaries and appreciating the time and effort required to communicate specialized knowledge. Finally, the project aimed to foster cross‐disciplinary trust and respect by prompting students to examine the assumptions they bring to others' work, reflect on how those assumptions shape their interpretations and interactions, and consider where their expectations may or may not align with how other disciplines actually operate. These aims are consistent with problem‐based learning, in which students develop knowledge and professional skills by working collaboratively on authentic, open‐ended problems (Lewinsohn et al. 2015), and with calls for conservation training that gives students direct practice navigating disciplinary differences (Andrade et al. 2014). Accordingly, the seminar centered on active ecological research questions and the iterative development of a usable proposal rather than on a hypothetical classroom exercise.

**FIGURE 1:**
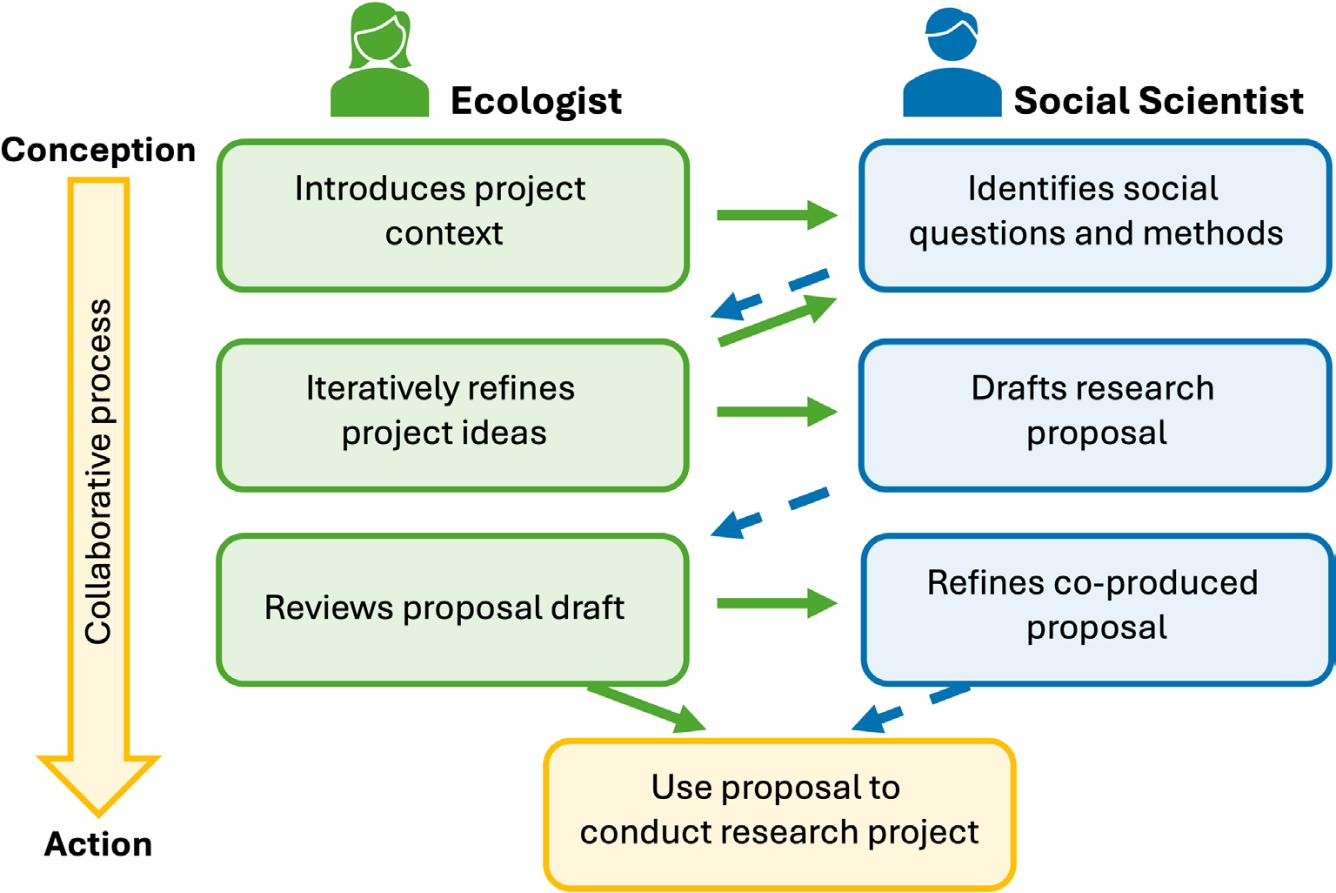
Over the course of a quarter or semester, ecology and social science graduate students work collaboratively to co‐produce a proposal focused on community‐engaged ecological research, which can be carried out in multi‐disciplinary teams. The proposal functions as a shared, revisable boundary object that coordinates exchange between the two disciplinary groups throughout the process.

The graduate course on qualitative methods was part of the Master's in Development Practice (MDP) program at Emory University in Atlanta, Georgia. Students were assigned readings on qualitative methods (Bartels and Furman 2024; Hennink et al. 2020; Stern 2018) as preparation for devising experiments with ecologists. Eight social science master's students from the MDP program were randomly assigned to two teams that collaborated with one of two ecology doctoral student volunteers. The ecology students both conduct research in the area of restoration ecology, were recruited by Dr. Jennifer Funk, and participated in the graduate seminar remotely from San Diego State University and the University of California, Davis. The seminar used a structured, semester‐long collaboration between social science and ecology graduate students to design research proposals focused on community engagement (Figure 1). In contrast to conventional research proposals, which are often organized around investigator‐defined questions, proposals for community‐engaged research emphasize collaboration with community partners in identifying relevant questions, shaping project goals, and considering how research outcomes may be useful beyond academic audiences. These projects may be developed as stand‐alone research proposals or incorporated into more conventional research proposals as broader‐impact activities. Community stakeholders were not directly involved in developing the proposals during the seminar; instead, the proposals outlined how relevant stakeholders would be engaged during subsequent refinement and, if the projects moved forward, implementation. The proposal format was chosen because it could be completed within a semester and because proposal documents are widely recognized as effective boundary objects for interdisciplinary collaboration and applied problem solving (Reilly et al. 2021; Star and Griesemer 1989). Boundary objects are shared tools that are adaptable enough to be meaningful within different disciplinary groups yet stable enough to coordinate work across them. In this seminar, successive proposal drafts served this function by giving ecology and social science students a concrete, revisable text around which to identify and negotiate differences in terminology, assumptions, research priorities, methodological choices, and feasibility.

Early in the term, ecology students prepared written overviews of their dissertation projects, including background, research questions, and key terminology intended to make their work accessible to non‐specialists. Using these dissertation overviews as a starting point, social science students then developed related community‐oriented research proposals as class projects. These proposals were distinct from the ecology students' dissertation projects but were designed to explore how the ecological research might be extended, translated, or reframed for community‐engaged contexts. Social science students received written feedback on these proposals from their ecology partners. Mid‐semester, two weeks after the proposals were submitted, the ecology students gave a formal presentation of their research via Zoom which allowed all course participants to ask questions, surface misunderstandings, and refine project designs. In the final half of the semester, the social science student groups met periodically with their ecology partner to refine the proposal. During the last week, the social science groups gave a formal presentation and submitted the final proposal, which was provided to the ecology students to support the funding and implementation of the project for community‐engaged research. Proposal development, rather than project implementation, was the course assignment. After the course, responsibility for pursuing each proposal rested with the associated ecology student and the relevant project team.

## 3 Proposed Projects

The course culminated in the development of two research proposals, one developed by each student team. One project centered on developing an assessment tool for a science‐based elementary school course for 10‐year‐old students. The program uses a hands‐on, place‐based curriculum to build students' scientific literacy and capability, confidence, and sense of place. Students follow the scientific method by investigating how plant growth is affected by two soil types: potting soil and serpentine soil (a specialized soil found at nearby McLaughlin Natural Reserve). The program culminates in a field day at McLaughlin Natural Reserve, where students rotate through hands‐on ecology stations led by undergraduate and graduate students from University of California, Davis. Program coordinators and teachers have anecdotally observed increased engagement from previously apathetic students, along with improved understanding of local ecosystems (Brooke Wainwright, communication with teachers). The research proposal, developed collaboratively by ecology and social science students, included both an annual assessment and a one‐time, holistic impact assessment. The annual assessment aimed to evaluate the program's effect on students' (a) understanding of the scientific method, (b) sense of capability, (c) sense of identity and place, and (d) interest in pursuing science, technology, engineering, and mathematics (STEM) studies or careers, using a pre‐ and post‐course “sticker” survey in which students place a sticker along a visual continuum from disagreement to agreement in response to relevant statements. MDP students also proposed incorporating classroom and field day observations to capture student involvement and reactions. The holistic impact assessment would engage key groups such as the teacher, former participants, the principal, classroom volunteers, and program coordinators through semi‐structured interviews and focus groups. Overall, the student researchers anticipated increased appreciation of local environments, stronger confidence and competency in science among participating elementary students, and positive impacts among stakeholders. The first project is now being carried out by ecology student participant Brooke Wainwright. Data were collected in spring 2026, and the results will be analyzed and reported in a subsequent paper.

The second project aimed to gather data on how attitudes toward restoration ecology methods and goals are changing among researchers versus practitioners in the United States, drawing on their lived experience. Students asked: what factors shape restoration strategies for researchers compared with practitioners, and why? They were also interested in how open each group is to prioritizing restoration of ecosystem function, rather than the more common goal of restoring historic species composition. The project proposed using qualitative surveys, in‐depth interviews (IDIs), and focus group discussions (FGDs) to better understand the complex links between human activities and restoration, and how researchers and practitioners perceive the restoration process. The survey would help identify broad patterns in restoration work (e.g., ecosystem types, positionality, years of experience), while the IDIs and FGDs would capture the experiences and values that inform decision‐making. Ultimately, this project seeks to illuminate shifting trends in restoration ecology amid rapid global change, while recognizing the nuanced, context‐dependent choices that both practitioners and researchers must make to achieve their restoration goals. The second project remains at the proposal stage.

## 4 Student Reflections

Throughout the semester, students kept reflective notes on their experiences of interdisciplinary collaboration, expectations, challenges, and strategies for navigating frustrations, following best practices in community‐engaged and qualitative research pedagogy. The guiding questions provided by the instructors are presented in Table 1. At the end of the course, students synthesized these reflections within their disciplinary teams, and the instructors compiled and organized the insights into a co‐authored account of the seminar and its outcomes (Furman et al., unpublished data). For this case study, we summarize recurring observations concerning three aspects of the seminar experience that relate directly to its aims: the practical work of communicating across disciplines, differences in methodology, and the timing and sustained engagement required for meaningful co‐design.

**TABLE 1:** Instructors provided the students four guiding questions for reflection as they navigated collaboration, expectation, and challenges in the semester‐long graduate seminar.

What were your expectations for working across disciplines at the beginning of the project? Summarize your general experience of working across disciplines. What aspect/s of this project surprised you? What aspect/s of this project proved most frustrating? How and to what extent where you able to navigate frustrations? In what ways will this experience impact how you conduct or envision multi‐disciplinary research in the future? How can we create a better classroom project moving forward?

*Note:* Key themes from student responses are summarized in the “Student reflections” section. A fuller qualitative analysis will be presented in Furman et al. (unpublished data).

Across both disciplinary teams, students described the collaboration as a meaningful (and at times humbling) introduction to what “working across fields” actually entails in practice. Social science students emphasized the excitement of contributing to real ecological work and the sense that they were helping lay groundwork for co‐production, and they reported feeling accomplished by the end of the process as they gained familiarity with restoration ecology concepts and with the role of social scientists in restoration research. At the same time, both groups noted that early stages of the collaboration were slowed by virtual logistics (including time zone differences) and by mutual misunderstandings rooted in unspoken assumptions about what the other group already knew. As one social science student group wrote, “The work was done virtually. The time difference caused students to hold meetings in the evening which ran late, disrupting personal and school activities.” From the ecology students' perspective, “Establishing a basic understanding of the other's work took considerable time and numerous, sometimes intense, conversations,” and “even the most basic terminology became confusing because of the multiple meanings across the two disciplines.” Students described having to “translate” not just terminology, but also the context and priorities that make a project feasible within each discipline, and both teams reported that progress accelerated only after they began asking more questions, doing background reading outside their discipline, and treating confusion as a signal to slow down and build shared understanding.

Reflections also highlighted deeper differences in norms and methodology across disciplines that shaped how students evaluated proposed designs. Ecology students noted that seemingly straightforward ecological terms could be “loaded” or contested in social science contexts, and they sometimes felt hesitant about how to frame ideas (e.g., demographic grouping, socioeconomic status, or generational differences) for fear of using language that could be interpreted as biased or inappropriate. For example, one ecology student wrote that “terms common in ecology (e.g., ‘historical baseline’) suddenly became contentious because in the social sciences it could be considered a ‘loaded term’.” Another described feeling uncomfortable discussing the socioeconomic context of a project site, writing, “I felt as though I could be judged for using incorrect language.”

Social science students, in turn, described moments of pushback when defending qualitative or mixed‐methods choices that differed from what their ecology partners were accustomed to, and some felt their expertise was not fully heard in early drafts. Social science students described this tension: “There was some pushback with the [ecology] student on the methods we proposed. We had to explain and defend why our methods would be more suitable than the methods she might be used to.” They also reflected that their “ideas were not heard,” despite their training in research methods and data collection tools. Both teams pointed to the importance of instructor facilitation: students repeatedly described the instructor's role as a “bridge” that helped translate expectations and navigate power dynamics. By the end, students reported a stronger appreciation for the labor and value of the other discipline, and several noted that productive interdisciplinary work requires not just division of labor (“the ecologist” versus “the social scientist”), but a shared vocabulary and unified vision built through sustained, structured interaction. These accounts speak directly to the course's aim of fostering cross‐disciplinary trust and respect by prompting students to recognize and reconsider assumptions about one another's expertise and ways of working.

Finally, ecology students repeatedly identified timeline and sequencing as a central lesson from the seminar. Through the seminar, students came to recognize that meaningful collaboration, relationship‐building, and qualitative or mixed‐methods design require substantial time and sustained engagement, and that social science expertise should not be treated as an “add‐on” late in a project. This recognition led them to conclude that if ecological research aims to generate socially relevant outcomes, social scientists should be integrated early, during problem framing and study design, rather than brought in only after key decisions have been set or projects are nearing completion. Taken together, the reflections suggest that students came to understand interdisciplinary co‐design not as a simple division of disciplinary tasks, but as an iterative process of building shared understanding and integrating different forms of expertise during problem framing and study design.

## 5 Lessons Learned and Recommendations

Preparing emerging ecologists to address critical policy, management, and conservation challenges requires more than a solid understanding of disciplinary content; it also demands practice in collaborative, cross‐disciplinary problem solving (Andrade et al. 2014). Many of the competencies built through authentic research experiences are the same ones that students need to succeed in academic, governmental, and other professional pathways. Expanding access to these practice‐based learning opportunities should therefore be a central goal of ecology education (Ban et al. 2015; Lewinsohn et al. 2015), including in courses and seminars that have shifted to remote or hybrid formats (Gahl et al. 2021).

One key lesson from this course was how difficult it can be for students to understand one another across disciplinary lines. Although each team's experience was distinct, both ecology and social science students described similar frustrations and ultimately worked to turn those moments of confusion into learning opportunities. This concurs with other findings that collaborations between ecologists and social scientists are often hindered by different terminologies, methods, and underlying philosophical assumptions, and that building shared analytical frameworks and making those assumptions explicit are critical steps toward more effective interdisciplinary work (Lélé and Norgaard 2005; Phillipson et al. 2009). Notably, students also described “wins” that emerged from these early stumbling blocks: ecology students reported gaining new appreciation for the rigor and time demands of qualitative social science research, while social science students noted how much ecological background knowledge was required to fully understand the scope of the scientific questions.

To better support that learning curve in future iterations, we recommend scaffolding early cross‐training through short, targeted readings that introduce each group to the other's core vocabulary, research logics, and common workflows. For example, instructors could provide a shared topical “theme” and a small set of background readings that build ecological context for social science students, while also assigning brief primers that help ecology students interpret qualitative and mixed‐methods approaches. Structured in‐class activities (e.g., paired mini‐presentations, annotated exemplars, or small‐group “teach‐back” exercises) could further reinforce this exchange, with student teams iteratively refining their proposals while explicitly articulating assumptions, definitions, and methodological choices (Fiorella and Mayer 2013). Because instructors may emphasize different qualitative approaches, reflecting the diverse philosophies and methodological traditions of the social sciences (Moon et al. 2019), the specific readings and exemplars could be tailored accordingly, while preserving the broader goal of helping students learn to translate across disciplinary boundaries.

In addition to assigned readings, we would make several changes to the course structure to give ecology students repeated practice in jointly framing research questions, negotiating methodological choices, and interpreting evidence with social scientists. In the current format, ecology students proposed the research questions and social science students developed the study design and methods. In future iterations, students from both groups would co‐generate questions and approaches through a more iterative process (Figure 1), dedicating several class meetings to refining the project framing, context, and feasibility. We would also clarify responsibilities for data analysis and interpretation. Rather than handing ecology students a proposal with no clear plan for who analyzes the data, future proposals would specify analytic roles and create opportunities for social science students to remain involved if the project moves forward. This may be especially critical when proposed research involves human subjects methods (e.g., surveys or in‐depth interviews) that require institutional ethics review and approval and specialized training that ecology students may not have.

Because the seminar was conducted virtually across institutions with different time zones and academic calendars, students had fewer opportunities for real‐time interaction in which disciplinary assumptions could be surfaced, questioned, and negotiated, and instructors had fewer opportunities to model that process. Ideally, this seminar would be offered within a single institution with regular in‐person meetings, which would reduce those coordination barriers and allow more sustained instructor involvement. However, cross‐institutional virtual collaborations can still succeed if they are intentionally structured, with more frequent, scheduled touchpoints and clear coordination throughout the term. In future iterations of this course, we aim for instructors to share the classroom more evenly so that students have consistent access to expertise from both disciplines as they navigate cross‐cutting questions. More balanced instructor involvement would also help faculty bridge disciplinary divides, validate students' questions and emerging interpretations, and model how to work productively across institutional and epistemic boundaries.

Effective responses to complex environmental challenges depend on collaboration between natural and social scientists, yet early‐career researchers rarely receive explicit training in the skills that such collaboration demands (Kelly et al. 2019; Record et al. 2016). The seminar described here illustrates how project‐based, multi‐disciplinary graduate courses, grounded in collaboration and co‐production, can begin to fill that gap. Although our example centered on plant and restoration ecology, this format could readily be applied to a wide range of issues in ecology and conservation. In line with recent calls to use graduate training, workshops, and other mechanisms to build capacity across institutional and sectoral boundaries (Bufford et al. 2025), this model treats listening across disciplines, navigating discomfort, and collaborating with humility and curiosity as core professional competencies rather than optional “soft skills.” By adopting and adapting similar approaches, educators can better equip the next generation of ecologists to translate their research into meaningful, real‐world solutions.

## Author Contributions

**Jennifer L. Funk:** conceptualization (equal), project administration (supporting), writing – original draft (lead). **Carrie A. Furman:** conceptualization (equal), project administration (lead), writing – review and editing (supporting). **Elizabeth M. Becker:** investigation (equal), writing – review and editing (supporting). **Brooke E. Wainwright:** investigation (equal), writing – review and editing (supporting). **Sandra N. Amoh:** investigation (equal), writing – review and editing (supporting). **Sameen Ahmed:** investigation (equal), writing – review and editing (supporting). **Irene A. Darkwaah:** investigation (equal), writing – review and editing (supporting). **Mariana Cubillos:** investigation (equal), writing – review and editing (supporting). **Hannah Jose:** investigation (equal), writing – review and editing (supporting). **Sandra E. Kotoka:** investigation (equal), writing – review and editing (supporting). **Meleanitema Sapoi‐Finau:** investigation (equal), writing – review and editing (supporting).

## Funding

This work was supported by National Institute of Food and Agriculture, 2021‐67019‐33429; U.S. Department of Agriculture, CA‐D‐PLS‐2247‐H.

## Conflicts of Interest

The authors declare no conflicts of interest.

## Data Availability

No primary data is included in this manuscript.
